# High-throughput profiling of the T cell receptor delta CDR3 repertoire reveals species-specific patterns in cattle (Bos taurus) and water buffalo (Bubalus bubalis)

**DOI:** 10.3389/fimmu.2026.1832949

**Published:** 2026-05-28

**Authors:** Yueheng Zhang, Fengli Wu, Long Ma, Xinsheng Yao, Jun Li

**Affiliations:** 1 Department of Immunology, Center of Immunomolecular Engineering, Innovation & Practice Base for Graduate Students Education, Zunyi Medical University, Zunyi, China; 2 Department of Pathology, and Precision Medicine Center, Yaan People’s Hospital (Yaan Hospital of West China Hospital, Sichuan University), Yaan, China

**Keywords:** cattle, TCR CDR3 repertoire, V(D)J recombination, water buffalo, γδ T cells

## Abstract

**Background:**

γδ T cells constitute a substantial proportion of lymphocytes in ruminants, and the diversity of their immune receptors is critical for understanding species-specific immune functions. The complementarity-determining region 3 (CDR3) of the T cell receptor delta chain (TRD) is a key structural determinant of γδ T cell antigen recognition; however, systematic comparative analyses of TRD immune repertoire characteristics across different bovine species remain limited. In this study, high-throughput sequencing and comprehensive analysis of the TRD CDR3 immune repertoire were performed in 7 cattle (Bos taurus) and 5 water buffalo (Bubalus bubalis).

**Results:**

The results demonstrated good consistency in sequencing depth and data quality between the two groups. Diversity analysis revealed that the cattle TRD CDR3 repertoire exhibited higher clonal evenness and overall diversity than that of buffalo. Clonotype composition analysis showed that both species were dominated by medium- to high-frequency clonotypes, whereas buffalo relied more heavily on a limited number of highly expanded clonotypes. V(D)J gene usage analysis identified pronounced species-specific preferences in TRDV gene usage, with high intra-group consistency but low inter-species correlation; in contrast, TRDJ gene usage was highly conserved between the two species. The CDR3 length distributions in both groups displayed similar bell-shaped patterns, suggesting structural constraints during evolution, while K-mer and motif analyses revealed differences in CDR3 microstructural features between species. Furthermore, shared clonotype analysis indicated a limited number of public CDR3 amino acid sequences between cattle and buffalo, with highly abundant shared clonotypes enriched only in a small subset of individuals.

**Conclusions:**

Collectively, this study provides a systematic characterization of the similarities and differences in the TRD CDR3 immune repertoires of cattle and water buffalo, offering fundamental data and a comparative perspective for understanding the mechanisms shaping γδ TCR diversity and their potential immunological functions in high γδ T cell species.

## 1 Introduction

T cells are broadly classified into two major subsets, γδ T cells and αβ T cells, based on the composition of their antigen receptors. αβ T cells represent the central effector population of the adaptive immune system, whereas γδ T cells possess characteristics of both innate and adaptive immunity and are therefore regarded as “bridging cells” between the two immune arms (1). The γδ T cell receptor (TCR) enables γδ T cells to recognize non-peptidic and stress-induced antigens in a major histocompatibility complex (MHC)-independent manner, allowing them to function as rapid immune sentinels at the interface of innate and adaptive immunity (2–4). Although γδ T cells constitute a minor fraction of peripheral lymphocytes in most species, they represent a dominant lymphocyte subset in cattle, accounting for approximately 60% of circulating T cells in neonatal calves (2). This striking species-specific difference in γδ T cell abundance suggests that bovine γδ T cells may fulfill immune surveillance and defense functions distinct from those observed in other mammals. The functional competence of γδ T cells is largely dependent on their TCR repertoire, particularly the highly diverse complementarity-determining region 3 (CDR3) of the δ chain (TRD), which plays a critical role in antigen recognition and the sensing of tissue stress signals (5, 6).

With the advancement of high-throughput sequencing (HTS) technologies, research focusing on the γδ TCR repertoire, especially the CDR3 repertoire of the TRD, has garnered increasing attention (7, 8). At the functional level, the length, sequence composition, and junctional diversity of the TRD CDR3 region are recognized as critical determinants governing the antigen recognition specificity of γδ T cells. The TRD CDR3 regions of ruminants typically exhibit greater length and structural diversity. Moreover, their V–D–J recombination patterns and nucleotide insertion/deletion profiles exert a profound impact on the spatial conformation of CDR3, thereby shaping a unique antigen-binding interface (7). In humans, the diversity of the TRD CDR3 repertoire is markedly higher than that of the TCR γ chain (TRG) repertoire, with a low degree of sharing among individuals, which reflects the central role of the δ chain in the functional differentiation of γδ T cells (5). Furthermore, the V, D, J gene usage and CDR3 characteristics of the TRD repertoire undergo dynamic alterations across distinct developmental stages and immune statuses, indicating that the TRD repertoire is synergistically regulated by both developmental programs and antigen selection pressure (9, 10). Recent studies have further demonstrated that the characteristics of the TRD repertoire hold substantial biological significance in tumor-associated immunity (11, 12).

Although preliminary investigations into the TRD repertoire have been conducted in humans, mice, sheep, camels, and other species (13, 14), research focusing on the TRD CDR3 diversity, V(D)J gene usage characteristics, and repertoire structural divergence remains remarkably limited in bovids-particularly in cattle and buffalo, two species that exhibit high similarities in γδ T cell proportions, immune niches, and evolutionary backgrounds yet possess distinct phylogenetic differences. Importantly, the genomic organization of the TRD locus is highly conserved between cattle and buffalo, both harboring three tandem D-J-C clusters and a single inverted TRDV3 gene, with high sequence similarity in TRDJ and RSS elements (93.9%–100% for TRDJ) and a shared expansion of the TRDV1 family, albeit with different copy numbers (52 in cattle vs. 61 in buffalo)(Additional file 1). Therefore, systematic characterization of the TRD repertoire features in cattle and buffalo will not only deepen the understanding of γδ T cell immune diversity and evolutionary rules in ruminants but also provide critical fundamental data to elucidate the specific immune functions of γδ T cells in species with high γδ T cell abundances.

## 2 Materials and methods

### 2.1 Sample collection and DNA extraction

We collected a total of 12 samples in this study, including spleen tissues from 5 healthy adult buffalo and 7 healthy adult cattle. All animals were sourced from a commercial abattoir located in Nanning, Guangxi, China, and had passed routine veterinary inspection prior to slaughter, indicating an absence of overt disease. Based on information provided by the abattoir, all animals were adult females approximately 2–4 years of age. Immediately after collection, the spleen tissues were snap-frozen in liquid nitrogen to prevent degradation. Genomic DNA was extracted using the DNeasy Blood & Tissue Kit (QIAGEN) following the instructions of the manufacturer. We evaluated the integrity, concentration, and quality of the extracted DNA via 1% agarose gel electrophoresis and an Agilent 2100 Bioanalyzer. Then, Qualified DNA samples were aliquoted and stored at -20 °C until subsequent experiments.

### 2.2 Primer design and synthesis

Based on the annotation results of the TRD locus, we designed amplification primers targeting the TRD CDR3 repertoire for cattle and buffalo, respectively. All forward primers were anchored in the conserved terminal regions of TRDV genes; a total of 5 gene-specific forward primers were designed, corresponding to the TRDV1-TRDV5 subgroups. Reverse primers were targeted to the conserved terminal regions of TRDJ genes, with 4 gene-specific reverse primers constructed to match the TRDJ1-TRDJ4 segments. All primers were synthesized by Sangon Biotech (Shanghai) Co., Ltd. Detailed sequences of the amplification primers are provided in Additional file 2.

### 2.3 TRD CDR3 repertoire construction and sequencing

Multiplex Polymerase Chain Reaction (PCR) was performed to amplify the target TRD CDR3 fragments. We prepared the PCR reaction system with a total volume of 50 μL, containing 25 μL of PCR Master Mix, 2.8 μL each of the forward and reverse primer mixtures, 1μg of genomic DNA template, and the remaining volume was supplemented with ddH_2_O. The PCR protocol was set as follows, with an initial denaturation step at 94 °C for 3 min, followed by 35 cycles of denaturation at 94 °C for 1 min, annealing at 56 °C for 1 min and extension at 72 °C for 1 min, then a final extension at 72 °C for 10 min and subsequent incubation at 4 °C. The PCR products were analyzed by 2% agarose gel electrophoresis to assess the brightness and specificity of the target bands. The purified and recovered target fragments were sent to BGI Genomics Co., Ltd (BGI Genomics) for quality inspection. Library preparation and sequencing were performed by BGI Genomics using the DNBseq platform. Briefly, purified PCR products were end-repaired, A-tailed, and ligated with sequencing adapters, followed by PCR amplification to construct sequencing libraries. The libraries were then circularized to form single-stranded DNA circles, which were subsequently amplified into DNA nanoballs. Sequencing was performed using a paired-end 150 bp strategy.

### 2.4 HTS data analysis of the TRD CDR3 repertoire

Raw sequencing reads were subjected to quality control prior to downstream analysis. Low-quality reads, reads containing ambiguous bases, and adapter-contaminated sequences were removed using standard filtering criteria. Quality metrics including Q20 and Q30 scores were evaluated to ensure high sequencing accuracy. Clean reads were then used for subsequent alignment and clonotype analysis. We performed quality control, sequence alignment, and clonotype assembly using MiXCR (v4.7.0) with default parameters optimized for T-cell receptor repertoire analysis. We uploaded the data in FASTQ format to MiXCR and aligned the clean sequencing reads against the species-specific background reference library covering the V, D, J, and C gene segments of the TRD locus (Additional file 3). Alignment was carried out using default alignment sensitivity without additional penalties for partial mapping, and clonotype assembly was performed using standard assembly stringency to group reads into unique CDR3 clonotypes, with no additional denoising steps applied to retain native clonotype diversity. In the Excel-formatted TRD CDR3 clonotype sequences output by MiXCR for each sample, first remove the unproductive sequences containing “*” or “_” in the “aaSeqCDR3” column. Then filter out sequences where the CDR3 amino acid sequence does not start with cysteine (C) and end with phenylalanine (F), thereby obtaining all fully functional TRD CDR3 sequences that are already confirmed to be in−frame and without stop codons.

### 2.5 Clonotype proportion distribution and diversity analysis

All statistical analyses were conducted in the R 4.5.2 software environment, using the immunarch (v0.9.0), ggpubr (v0.6.0), ggplot2 (v3.5.0), and UpSetR (v1.4.0) packages. Corresponding analysis scripts are deposited in Zenodo at DOI: 10.5281/zenodo.19510293. Firstly, we calculated the number of unique clonotypes for both the cattle group and buffalo group, followed by the calculation of the proportions of top clonotypes and rare clonotypes. A clonotype was defined as a set of sequences sharing an identical TRD CDR3 amino acid sequence. Unique clonotypes refer to the total number of distinct CDR3 amino acid sequences observed in a sample. Rare clonotypes were defined based on absolute read counts, categorized into groups of clones with 1, 3, 10, 30, and 100 reads using.method = “rare” (default.bound = c (1, 3, 10, 30, 100)). Top clonotypes were defined as the sets of the 10, 100, and 1,000 most abundant clonotypes per sample, and their cumulative relative abundances were calculated using.method = “top” (default.head = c(10, 100, 1000, 3000, 10000, 30000, 1e+05)). Public clonotypes were defined as CDR3 amino acid sequences shared by at least two individuals (either across all samples or within a species), whereas private clonotypes were those detected in only one individual. To comprehensively evaluate the diversity of the TRD repertoires between the two groups, multiple diversity metrics were used for further analysis, including D50 index, Gini-Simpson index and Hill index, so as to fully assess the richness and evenness of clonotypes in the two groups.

### 2.6 V, D and J gene usage and correlation analysis

Based on the data generated by MiXCR, the usage frequencies of V, D and J genes in each sample were calculated and expressed as percentages. For genes with significant differences, pairwise comparisons were performed using the Kruskal-Wallis H test, with P < 0.05 considered statistically significant. Subsequently, we analyzed the correlation of V, D and J gene usage within and between groups, applying the Spearman correlation coefficient to measure these associations and plotting heatmaps for visualization. The heatmaps were displayed according to the gradient of correlation coefficient values, which intuitively reflected the similarities in gene usage patterns between the two species and among samples.

### 2.7 CDR3 amino acid length distribution

Leveraging the data generated by MiXCR, we extracted all valid TRD CDR3 amino acid sequences and determined the amino acid length of each individual sequence. To minimize the impact of extreme values, only CDR3 sequences with lengths spanning 10 to 40 amino acids were retained, a filtering step that ensured the reliability of subsequent analyses. We quantified the distribution frequencies of CDR3 amino acid lengths across the two groups and then constructed length distribution histograms to visualize the variation trends directly. By comparing the peak values and distribution ranges of CDR3 lengths between the two groups, the fundamental structural characteristics that distinguish the CDR3 sequences of the two species were clearly revealed.

### 2.8 K-mers and motif analysis

To further explore the characteristic differences in CDR3 amino acid sequences, we selected 5-amino-acid short peptide fragments for analysis, counted the top 10 high-frequency fragments in each group, calculated their respective frequencies, and finally plotting stacked bar charts to compare inter-species disparities. Subsequently, motif analysis was performed based on amino acid composition, with the maximum number of motifs set to 5. By integrating the physicochemical properties of amino acids, such as hydrophobicity and polarity, we analyzed the composition characteristics, occurrence frequencies, and arrangement patterns of amino acids in different motifs, aiming to explore the potential association between species-specific motifs and immune functions.

### 2.9 TRD CDR3 repertoire overlap analysis

To evaluate the overlap of TRD CDR3 repertoires among the 12 samples, we quantified the number of shared clonotypes, and further calculated the proportion and quantity of shared clonotypes between each pair of samples. Thereafter, we plotted a heatmap of inter-sample overlap to visually display the shared characteristics of clonotypes within and between groups. With UpSet plots generated via the UpSetR package, we presented the number of specific clonotypes unique to the cattle group, the buffalo group, and those common to both in the form of waterfall charts. In this way, the uniqueness and commonality of TRD repertoires between the two species were clearly identified, thereby providing evidence for revealing species-specific immune characteristics.

### 2.10 Shared CDR3 sequence analysis across samples

Three categories of amino acid sequences were selected for comparative analysis, including the 5 CDR3 amino acid sequences shared most frequently across all samples, the top 5 sequences with the highest shared abundance within the buffalo group, and the 5 most prevalent shared sequences in the cattle group. We quantified the clonal frequency and proportion of each target sequence in the corresponding sample set, and plotted bar charts to compare the expression discrepancies of these sequences across different samples. By analyzing the conserved characteristics and variation patterns of CDR3 sequences between the two species, this study provides a molecular basis for clarifying the mechanisms underlying species-specific immune responses.”.

## 3 Results

### 3.1 Data processing and quality control of TRD CDR3 repertoires

Raw sequence data of TRD CDR3 repertoires from 12 samples (7 cattle samples and 5 buffalo samples) were processed, with sequence alignment and quality control carried out using MiXCR. After a series of processes including low-quality sequence filtering and sequence trimming, high-quality CDR3 clonotype data were generated and used for subsequent analyses (**Table 1**).

**Table 1:** Summary statistics of TRD CDR3 repertoire for individual buffalo and cattle samples.

Species	Sample	Total reads	Successfully aligned reads	Reads used in clonotypes	Final clonotype count	TRD chains
Cattle	C1	8154462	7423646 (91.04%)	5654847 (69.35%)	369414	368360 (99.71%)
	C2	8107152	6099183 (75.23%)	4119621 (50.81%)	190206	189961 (99.87%)
	C3	8137606	6608920 (81.21%)	4924724 (60.52%)	406037	405571 (99.89%)
	C4	8119303	7440230 (91.64%)	6297407 (77.56%)	374205	373569 (99.83%)
	C5	8063013	7277426 (90.26%)	6247908 (77.49%)	446402	445883 (99.88%)
	C6	8031451	7447628 (92.73%)	5755381 (71.66%)	161129	160483 (99.6%)
	C7	8116415	6568898 (80.93%)	4803053 (59.18%)	402179	400587 (99.6%)
Buffalo	B1	8115425	5401709 (66.56%)	3100477 (38.2%)	113358	113358 (100%)
	B2	8075301	6940735 (85.95%)	5726841(70.92%)	335655	335655 (100%)
	B3	8057834	4125104 (51.19%)	1901781 (23.6%)	165452	165452 (100%)
	B4	8003251	7016187 (87.67%)	5758459 (71.95%)	296555	296555 (100%)
	B5	8075327	6669232 (82.59%)	5784335 (71.63%)	442366	442366 (100%)

The TRD (T cell receptor delta) chain represents the delta chain of the γδ T cell receptor, generated by somatic rearrangement of TRDV, TRDD and TRDJ gene segments during T cell development; C denotes cattle samples (n=7); B denotes buffalo samples (n=5).

Raw sequencing reads of all samples exceeded 8,000,000, with the cattle group ranging from 8,031,451 to 8,154,462 and the buffalo group from 8,003,251 to 8,115,425, demonstrating comparable initial sequencing depth between the two groups. The final clonotype counts of the cattle group spanned from 161,129 to 446,402, while those of the buffalo group ranged from 113,358 to 442,366. A relatively large intra-group variation was observed, and the clonotype counts of the cattle group were slightly higher than those of the buffalo group.

“Notably, all TCR sequences obtained from buffalo samples were exclusively assigned to TRD chains (100%), whereas in the cattle group, the proportion of TRD chains ranged from 99.6% to 99.89%, with the remaining sequences corresponding to TRA chains. This discrepancy was primarily due to the difference in background reference libraries applied for the two groups: the reference library for buffalo was constructed in-house based on the germline genes annotated by our research team, while the library for cattle was directly adopted from the one provided by IMGT. Therefore, a small number of TRA chains were detected in the cattle group, which is consistent with the characteristic nested structure of the mammalian TRA/D gene locus where TRA and TRD chains are located on the same chromosome.

### 3.2 Clonotype and diversity analysis of TRD CDR3 repertoires

**Figure 1:**
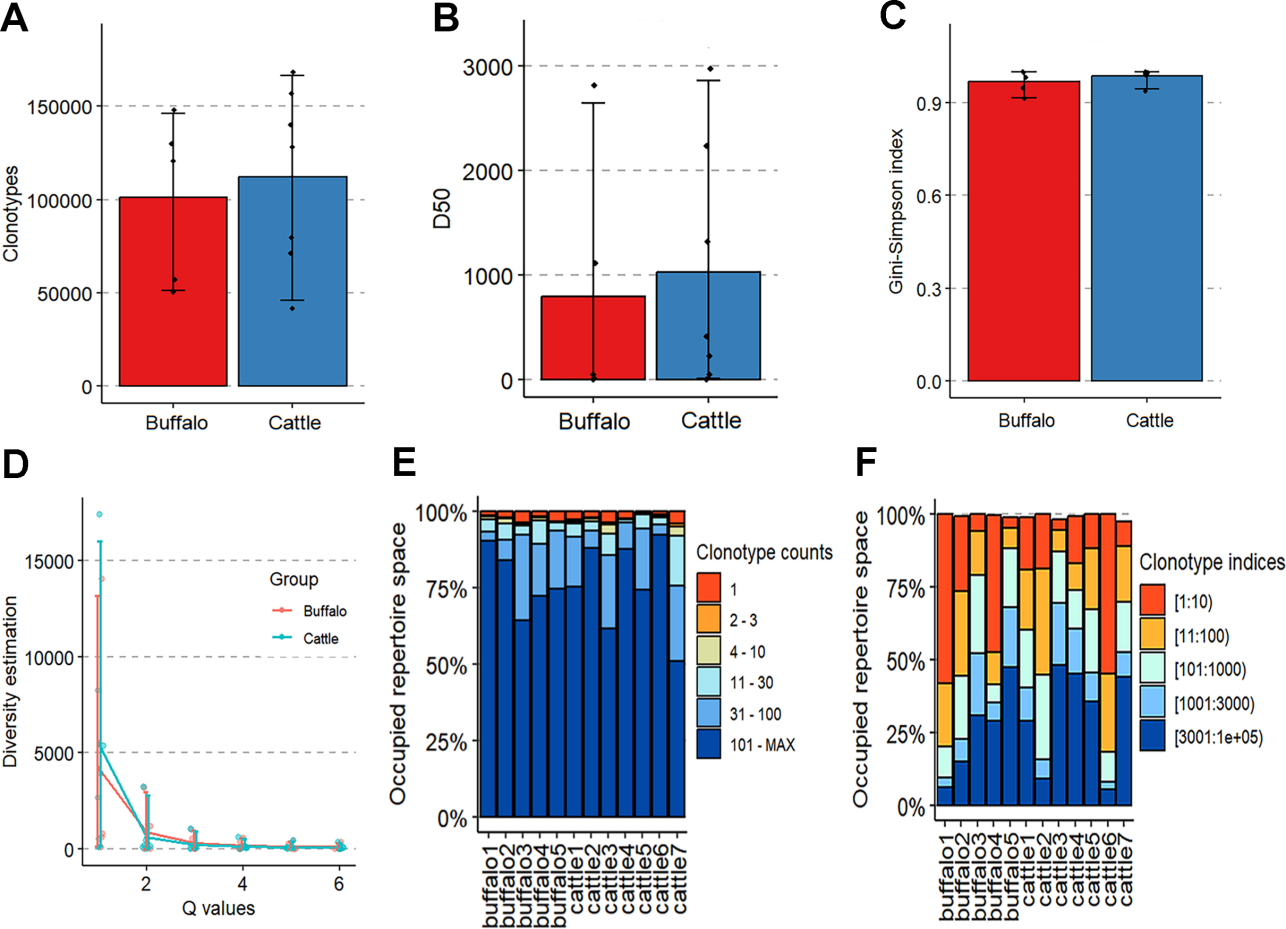
Clonotype and diversity of TRD chains between the cattle and buffalo groups.**(A)** Differential analysis of unique clonotypes between cattle and buffalo; **(B)** the estimation of repertoire diversity utilized in D50 index; **(C)** the estimation of repertoire diversity utilized in Gini-Simpson index; **(D)** the estimation of repertoire diversity utilized in Hill index; **(E)** the rare clonal proportion; **(F)** the top clonal proportion.

Statistical analysis of the unique clonotype counts in the buffalo and cattle groups showed that the cattle group had 71,990-170,067 unique clonotypes while the buffalo group had 51,202-150,363. The number of unique clonotypes in the cattle group was slightly higher than that in the buffalo group, with significant intra-group variation observed. However, no statistically significant difference was detected between the two groups (**Figure 1A**).The diversity of TRD CDR3 repertoires in the two groups was evaluated using three metrics, namely the D50 index(**Figure 1B**), Hill index(**Figure 1C**), and Gini-Simpson index(**Figure 1D**). The results consistently demonstrated that the TRD CDR3 repertoire diversity of the cattle group was higher than that of the buffalo group. Specifically, the higher D50 index of the cattle group indicated a more uniform clonotype distribution, while the Hill index and Gini-Simpson index further verified that the cattle group had a more even clonotype distribution and richer diversity.

Subsequent analyses were performed to characterize the proportion of rare clonotypes and top clonotypes in the two groups. For rare clonotypes, both the cattle and buffalo groups exhibited a low proportion of low-frequency clonotypes, with clonotypes present in fewer than 30 copies accounting for less than 10% of the total in each group(**Figure 1E**). Moreover, the two groups shared a highly similar distribution pattern of clonotypes across different abundance levels. Regarding top clonotypes, the top 10 most abundant clonotypes accounted for a higher proportion in the buffalo group than in the cattle group(**Figure 1F**), and the cumulative proportion of the top 100 clonotypes exceeded 50% in both groups (Additional file 4). These findings indicated that both buffalo and cattle repertoires were dominated by medium-to-high frequency clonotypes, with the buffalo group relying more heavily on a small number of highly abundant clonotypes. The result was consistent with the aforementioned conclusion, further confirming that the cattle group had a more uniform clonotype distribution and higher repertoire diversity.

### 3.3 V, D and J gene usage and correlation analysis of TRD CDR3 repertoires

**Figure 2:**
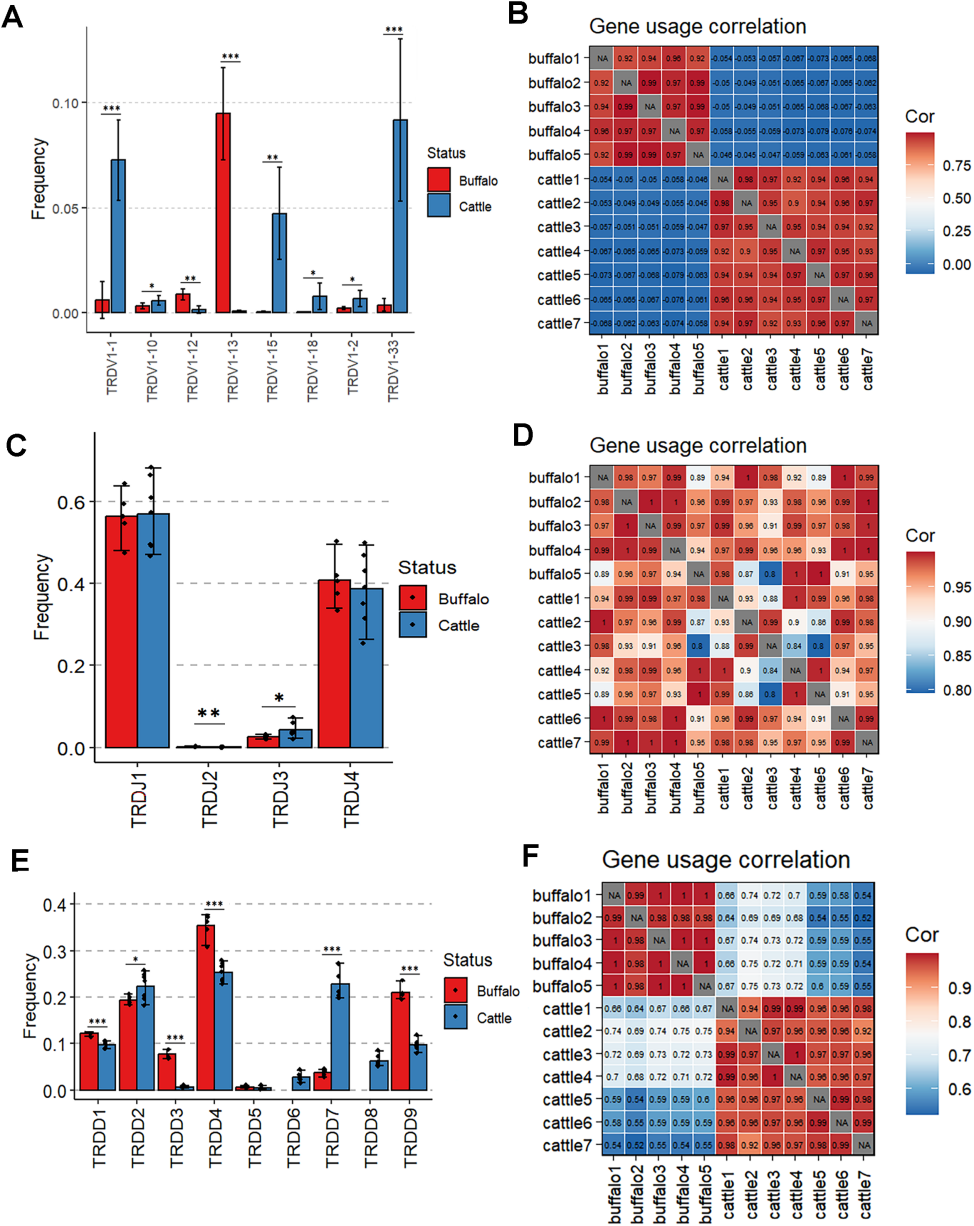
The usage and correlation of V, D and J genes of the TRD CDR3 repertoires. **(A)** statistical analysis of the V gene usage between the cattle and buffalo groups; **(B)** the correlation of V gene usage among all samples; **(C)** statistical analysis of the J gene usage between the cattle and buffalo groups; **(D)** the correlation of J gene usage among all samples; **(E)** statistical analysis of the D gene usage between the cattle and buffalo groups; **(F)** the correlation of D gene usage among all samples.(Kruskal-wallis H Test, * represents P<0.05, ** represents P<0.01, *** represents P<0.001).

IMGT has documented 55 TRDV genes in cattle, among which 52 were detected in this experiment. Meanwhile, 57 V genes were identified in this study out of the 65 annotated V genes in buffalo (Additional file 5). Analysis of V gene usage frequencies showed that most V genes were utilized at low frequencies. In the buffalo group, TRDV1–13 exhibited the highest usage frequency (nearing 10%), which was significantly higher than that in the cattle group. In contrast, the cattle group preferentially used TRDV1-1, TRDV1–15 and TRDV1–33 at high frequencies, with TRDV1–33 having the highest frequency (approximately 9%); the usage frequencies of these three genes were all significantly higher than those in the buffalo group. Several V genes, such as TRDV1-10, TRDV1-12, TRDV1–18 and TRDV1-2, were expressed at low frequencies in both groups, yet significant inter-group differences were still observed (**Figure 2A**). Collectively, V gene usage displayed distinct species-specific preferences between the two groups. Correlation analysis of V gene usage indicated a strong positive correlation among samples within each group, whereas the inter-group correlation was weak. These results demonstrated that V gene usage differed between groups and possessed species-specific characteristics (**Figure 2B**).

Four TRDJ genes of cattle have been recorded in IMGT, and our research team annotated four TRDJ genes in buffalo. The same four TRDJ genes were detected in both cattle and buffalo, and all of them were expressed across all samples. Analysis of J gene usage frequencies revealed that both groups predominantly utilized TRDJ1 and TRDJ4 at high frequencies. Among these TRDJ genes, TRDJ1 was the most frequently used (close to 57%), followed by TRDJ4 (close to 40%), with no significant differences in usage frequencies observed between buffalo and cattle groups. TRDJ2 and TRDJ3 were expressed at low frequencies in both groups but still showed significant inter-group differences (**Figure 2C**). Correlation analysis of J gene usage showed a strong positive correlation both within and between the two groups, suggesting that J gene usage was not species-specific and was highly conserved across species (**Figure 2D**).

Nine TRDD genes of cattle have been recorded in IMGT, and our research team annotated eight TRDD genes in buffalo (Additional file 3). Analysis of D gene usage frequencies revealed striking species-specific preferences between the two groups. In the buffalo group, TRDD4 exhibited the highest usage frequency (approximately 35%), followed by TRDD9 (around 21%). Notably, TRDD6 and TRDD8 were nearly undetectable in all buffalo samples, showing an almost complete absence of expression. In cattle, TRDD2 was also the most frequently used D gene (approximately 25%); while TRDD6 and TRDD8 were stably expressed in all cattle samples. Statistical analysis confirmed significant inter-group differences in the usage of most TRDD genes. Specifically, buffalo showed significantly higher usage frequencies of TRDD1, TRDD3, TRDD4, and TRDD9 compared to cattle, whereas cattle exhibited significantly higher frequencies of TRDD2 and TRDD7 (**Figure 2E**). Correlation analysis of D gene usage indicated a strong positive correlation among samples within each group, whereas the inter-group correlation was weak. These results demonstrated that D gene usage differed between groups and possessed species-specific characteristics (**Figure 2F**).

### 3.4 Length distribution, K-mers and motif sequence analysis of TRD CDR3 repertoires

**Figure 3:**
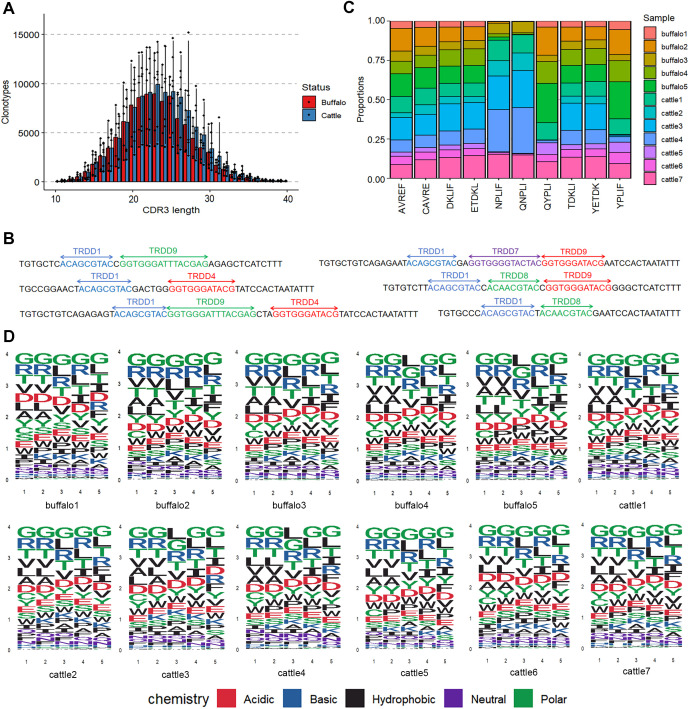
The length distribution, Kmers and motif sequences of the TRD CDR3 repertoires. **(A)** the length distribution of CDR3; **(B)** representative multi-D segment rearrangements in buffalo **(left)** and cattle (right); **(C)** the distribution of top 10 5-mers in sequence length; **(D)** position self-information matrix from sequence motif analysis.

Analysis revealed that the CDR3 length distribution of both groups exhibited a highly consistent bell-shaped pattern, with lengths mainly concentrated between 15 and 30 amino acids and a peak at 23 amino acids for each group. No significant difference was observed in clonotype abundance across different lengths between the two groups (**Figure 3A**). These results indicated that the CDR3 length distribution was conserved between the two species.

Notably, the CDR3 length of the TRD chain in both buffalo and cattle is generally longer than that of the TRB chain, which may be partly attributed to the presence of D-D fusion events. We analyzed D-D fusion segment rearrangements in both buffalo and cattle, and the successful identification of multiple D gene segments within a single CDR3 sequence directly confirmed the occurrence of D-D fusion in both species. Statistical analysis revealed a significant interspecies difference in D-D fusion frequency. In cattle, the proportion of CDR3 sequences derived from D-D fusion ranged from 1.90% to 5.58% across individual samples, with an average frequency markedly higher than that in buffalo. In contrast, D-D fusion events were much less prevalent in buffalo, with the proportion ranging from only 0.79% to 1.66% in all samples. Statistical analysis revealed a significant interspecies difference in D-D fusion frequency, with a higher proportion in cattle than in buffalo (Additional file 6). Further analysis showed that D-D fusion in both buffalo and cattle occurred with 2 or 3 D genes, with TRDD1 and TRDD9 being the dominant D genes in buffalo, and TRDD1 and TRDD8 in cattle (**Figure 3B**).

To further analyze the K-mers of the TRD CDR3 repertoires, the top 10 high-frequency 5-mer sequences were selected for frequency analysis based on K-mer occurrence counts. The results showed that the buffalo and cattle groups shared some K-mers while also displaying species-specific preferences. Among all 12 samples, DKLIF and TDKLI were the most frequently utilized K-mers. In the analysis of the top 10 high-frequency K-mers, two overlapping 5-mers (QYPLI and YPLIF, shifted by one residue) were predominantly present in the buffalo group, with each accounting for over 30% of total K-mer occurrences in buffalo5; by contrast, NPLIF and QNPLI were mainly detected in the cattle group, with each representing more than 30% of total K-mer occurrences in cattle4. The remaining K-mers showed minimal differences between the two groups (**Figure 3C**).

Subsequently, motif sequence analysis was performed based on amino acid composition. It was found that polar amino acids and hydrophobic amino acids exhibited relatively high frequencies in the core regions, which constituted the basic amino acid composition characteristics of CDR3 sequences. Amino acids including glycine (G), arginine (R), leucine (L) and valine (V)-which belong to polar, basic and hydrophobic categories-were detected in all samples, with G showing the highest frequency. In addition, the proportion of V at position 2 was higher in the buffalo group than in the cattle group, and individual differences were observed in the proportion of L at position 3 within the buffalo group (**Figure 3D**). These findings suggested that the order and arrangement of amino acid sequences differed significantly between species and among individuals.

### 3.5 Consensus amino acid sequences and overlap analysis of TRD CDR3 repertoire

**Figure 4:**
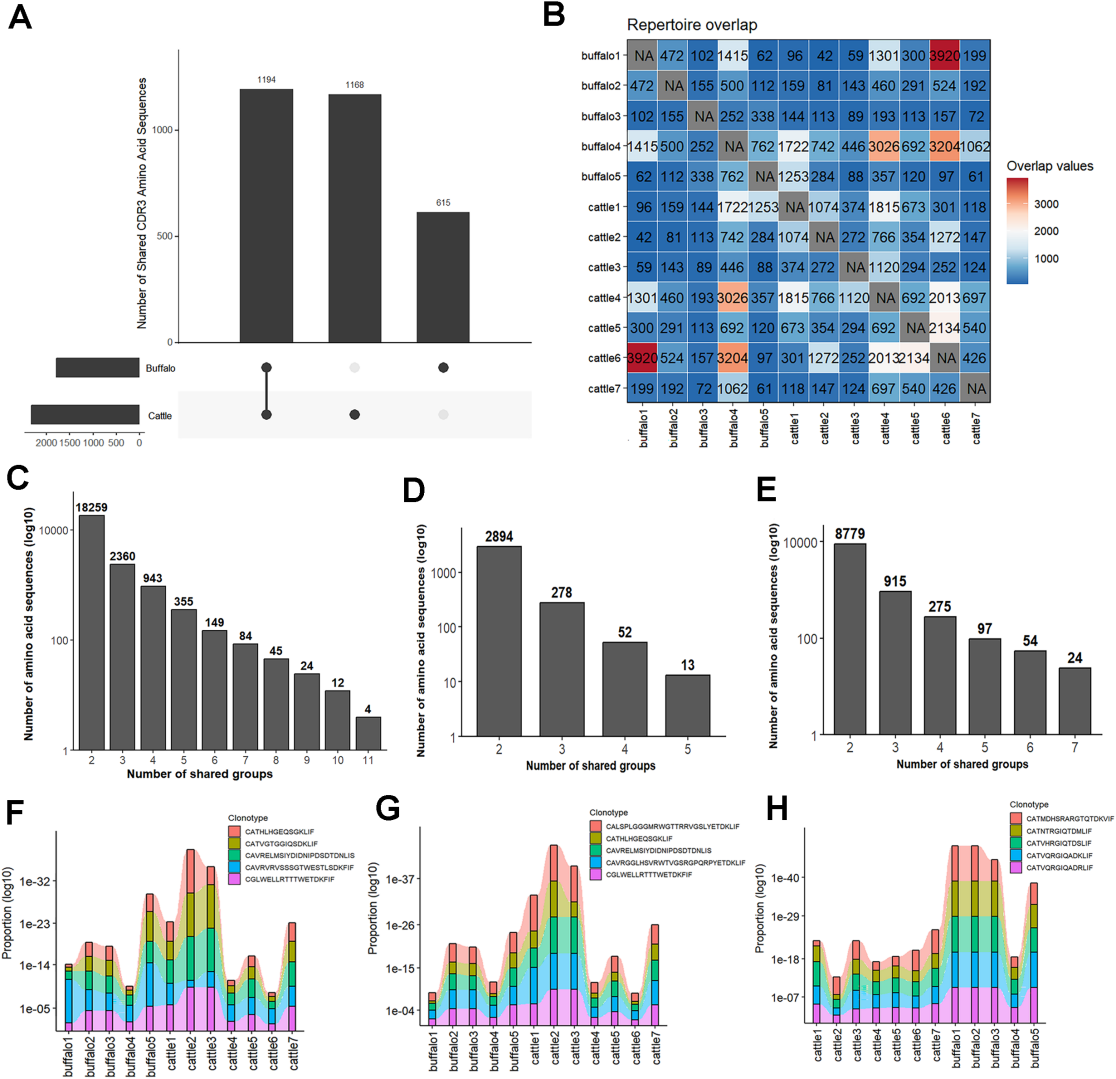
The shared amino acid sequence and repertoire overlap of the TRD CDR3 repertoires. **(A)** number of shared and amino acid sequences between buffalo and cattle; **(B)** heatmap of TRD repertoire overlap; **(C)** number of shared amino acid sequences among all samples across different numbers of shared groups; **(D)** number of shared amino acid sequences in the buffalo groups across different numbers of shared groups; **(E)** number of shared amino acid sequences in the cattle groups across different numbers of shared groups; **(F)** tracking analysis of shared clones among all samples; **(G)** tracking analysis of shared clones for the top 5 amino acid sequences in the buffalo groups; **(H)** tracking analysis of shared clones for the top 5 amino acid sequences in the cattle groups.

Analysis of conserved amino acid sequences in the TRD CDR3 repertoires of the two groups showed that 1,168 unique sequences were identified in the cattle group and 615 in the buffalo group, with 1,194 sequences shared between the two groups (**Figure 4A**). Analysis of conserved clonotype overlap revealed that the extent of clonotype sharing among individuals within the cattle group was higher than that within the buffalo group. For instance, the overlap values between cattle4 and cattle6, as well as between cattle5 and cattle6, exceeded 2,000. By contrast, only a few individual pairs, such as buffalo4 and buffalo1, exhibited relatively high overlap in the buffalo group. When comparing between the two groups, the overall overlap values were low, but several cross-group pairs, including buffalo1 and cattle6, buffalo4 and cattle6, and buffalo4 and cattle4, showed higher overlap values than those observed within either group (**Figure 4B**).

Analysis of shared amino acid sequences across samples demonstrated that 18,259 sequences were shared by 2 out of the 12 samples, whereas only 4 sequences were conserved across 11 samples (**Figure 4C**). Within the buffalo group, 2,894 sequences were shared by 2 samples, while merely 13 sequences were present in all 5 samples (**Figure 4D**). In the cattle group, 8,779 sequences were shared by 2 samples, and only 24 sequences were detected across all 7 samples (**Figure 4E**).

Tracking of the top five most frequently shared amino acid sequences across all samples indicated that buffalo1, buffalo4, cattle4 and cattle6 had relatively high numbers of shared sequences, with CATHLHGEQSGKLIF being the most prevalent shared sequence (**Figure 4F**). When focusing on the top five most frequently shared sequences within the buffalo group, buffalo1, buffalo4, cattle4 and cattle6 were again found to harbor abundant shared sequences, and CATHLHGEQSGKLIF remained the most dominant shared sequence (**Figure 4G**). For the cattle group-specific top five most frequently shared sequences, cattle1, cattle2 and buffalo4 showed high levels of sequence sharing, with CATMDHSRARGTQTDKVIF identified as the most commonly shared sequence (**Figure 4H**). These results suggested that highly abundant shared clonotypes were enriched only in a small subset of individuals.

## 4 Discussion

γδ T cells account for an extremely high proportion in ruminants, a feature that is significantly different from that observed in other γδ low species such as humans and mice. Early studies have indicated that γδ T cells in ruminants including sheep and cattle represent the dominant subsets in both peripheral blood and tissues, a characteristic that may reflect the unique surveillance requirements of the ruminant immune system in response to external stimuli (3, 4, 15). In addition, γδ TCRs are capable of recognizing non-classical antigens and acting as a bridge between innate and adaptive immunity, endowing these cells with a critical role in the rapid response to pathogenic challenges (3). This study systematically compared the characteristics of TCRδ CDR3 immune repertoires between two bovine species, cattle and buffalo. Multiple dimensions including clonal diversity, V(D)J gene usage, CDR3 structural features and shared clonotypes were investigated, which revealed remarkable differences between the two species against the backdrop of their highly similar γδ T cell populations. Overall, although both species exhibited a TRD CDR3 repertoire architecture dominated by medium-to-high frequency clonotypes, the clonotype distribution in cattle was more homogeneous, with an overall diversity level higher than that in buffalo. These observations suggest that γδ T cells of different bovine species may adopt distinct immune strategies to combat external antigen stimulation, even when they occupy similar immune niches.

The basic structure and composition of the TRD locus are the root causes of the complexity of the TRD repertoire. In bovids, the TRD gene pool is extremely large, the TRDV1 subgroup alone comprises 52 TRDV genes, along with 9 D genes and 4 J genes. This gene amplification phenomenon is prevalent in γδ−high species (7, 16). Specifically, the TRD locus exhibits abundant TRAV/TRDV members, including different subgroups such as TRDV1, TRDV2, TRDV3 and TRDV4, in the annotation of multiple genome versions, along with multiple TRDD and TRDJ segments. These provide a rich genetic basis for high CDR3 diversity (16, 17). Such gene amplification is not only prominent in cattle but also shows a similar pattern in other ruminants like sheep, indicating an evolutionary commonality in the topological expansion of TRDV1 among ruminant species (7). In this study, we observed that the cattle group used a total of 52 V genes, among which 47 belonged to the V1 family, accounting for 90.4%. In contrast, 57 V genes were detected in the buffalo group, with 53 from the V1 family, representing a proportion of 93.0%. This result further confirms the amplification characteristic of the TRDV1 subgroup in bovids. Notably, both the total number of TRDV genes used and the proportion of the V1 family in buffalo were slightly higher than those in cattle, suggesting that the TRD repertoire in buffalo may have richer diversity. Meanwhile, the V1 family genes dominated in both species, highlighting their important role in γδ T cell receptor assembly and conforming to the evolutionary common feature of TRDV1 topological expansion in ruminants. Additionally, studies have found that the expansion of TRDV1+ γδ T cells in ruminants may be attributed to the unique surveillance requirements of their immune systems (17).

In terms of V gene usage patterns, cattle and buffalo exhibited distinct species-specific preferences. Correlation analysis of TRDV usage between cattle and buffalo showed a high degree of consistency within each group but low correlation between the two groups, suggesting that TRDV gene usage is driven by species-specific regulatory mechanisms. Although both buffalo and cattle retain a large TRDV1 gene pool (17), the frequency and usage preference of their specific gene members are not completely consistent between the two species. This may reflect the different selective pressures experienced by the two species during long-term independent evolution and ecological adaptation. In addition, different V gene usage patterns may also be associated with their immune responses to the same pathogens and the outcomes of infection. In a Schistosoma japonicum infection model, after infection, cattle (Bos taurus) are more prone to developing hepatic white granulomas and significant inflammatory cell infiltration, whereas buffalo (Bubalus bubalis) exhibit relatively milder inflammatory responses (18). Similarly, during Mycobacterium bovis infection, although both species can produce antigen-specific IFN−γ and TNF−α cytokine responses, differences in cytokine expression patterns have been observed between buffalo and cattle, indicating interspecific variations in immune responses (19). Beyond these specific infection models, specie-specific differences in immune reactivity have also been observed following vaccination, where cattle often elicit stronger humoral and cellular responses than buffalo to the same vaccine formulations (20, 21). Additionally, husbandry factors such as nutrition, environmental stressors, and disease management strategies can profoundly influence immune competence, further shaping the immunological divergence between cattle and buffalo (22).

In contrast to the species-specific differences observed in V gene usage, TRDJ genes exhibited higher conservation between cattle and buffalo. Both groups predominantly used TRDJ1 and TRDJ4, with an extremely high correlation across the two species. Such conservation in J gene usage also shows similar cross-species stability in the β chain locus of αβ TCR. Previous immune repertoire and comparative genomics analyses have demonstrated that TRBJ segments in TCR β repertoires of multiple mammalian species, including bank voles, mice and humans, display significant sequence conservation and play a critical role in shaping the terminal structure of CDR3 (23–25). This conservation may be attributed to the functional constraints of J genes in forming the structural framework of the CDR3 terminal region after V(D)J recombination. Specifically, J segments provide the structural backbone of the CDR3 region, enabling the variable region of the entire receptor to stably bind to D/V segments and maintain the overall three-dimensional conformation. This functional characteristic is supported by comparative analyses of gene loci across various species (25). However, the use of multiplex PCR primers targeting conserved TRDV and TRDJ regions may introduce amplification bias among different V and J genes, which could potentially influence the observed gene usage frequencies.

The TRD CDR3 length distribution in both cattle and buffalo showed a typical bell-shaped pattern, with lengths concentrated in the range of 15–30 amino acids. This range is wider than that of the TCR β chain CDR3 in αβ high species such as humans and mice, which usually falls between 12 and 15 amino acids (26). This phenomenon may be attributed to the fact that the TRD gene pool of ruminants including cattle and sheep contains a large number of TRDV1 subgroups and multiple D/J genes, which provides a genetic basis for generating CDR3 regions with diverse and variable lengths (7, 16, 17). For example, in sheep, the usage of different numbers of TRDD genes directly leads to a linear increase in CDR3 length, with the longest CDR3 even exceeding approximately 26 amino acids (7). Similarly, we detected DD fusion events (two or three D genes in tandem) in both cattle and buffalo. However, if multiple D genes combine with extensive non−template insertions, the resulting CDR3 could become extremely long, pushing the amplicon beyond 300–350 bp, beyond the reliable coverage of our PE150 sequencing. This technical limitation warrants consideration in studies involving ultra−long γδ TCR CDR3s, for which long−read sequencing is recommended.

In this study, the number of shared amino acid sequences between the TRD CDR3 repertoires of cattle and buffalo was relatively small, and highly abundant shared clonotypes were restricted to a limited subset of individuals. Tracking of the top shared sequences revealed that clonotypes were repeatedly detected as dominant shared sequences, but their enrichment was limited to specific animals (e.g., buffalo1, buffalo4, cattle4, and cattle6), rather than being consistently distributed across all individuals. This pattern further supports the notion that γδ TCR repertoires are characterized by strong individual specificity, with public or shared clonotypes occurring only in a restricted subset of repertoires. For example, deep sequencing data have indicated that multiple public clonotypes exist in adult Vγ9Vδ2+ repertoires, whereas other γδ subsets such as Vδ1+ are dominated by private clonotypes (9, 27, 28). In addition, cattle (Bos taurus) and buffalo (Bubalus bubalis) both belong to the tribe Bovini, yet their genomes exhibit significant differences in phylogenetic history and chromosomal structure. Comparative genomics studies have shown that the two species have undergone multiple structural chromosomal rearrangement events since diverging from their common ancestor. These genomic changes help to explain the potential differences in rearrangement preferences near specific gene loci (29, 30). Finally, the divergence time between cattle and buffalo can be traced back to millions of years ago. The two species have experienced distinct natural and artificial selection pressures under their respective ecological and domestication backgrounds, which may fundamentally affect the rearrangement tendency and generation probability distribution of the TRD locus (31).

Buffalo and cattle, both belonging to Bovidae, are characterized by a γδ T cell dominant immune system (32) and exhibit markedly higher TRD repertoire complexity compared with humans and mice. These observations are consistent with recent evidence indicating that ruminant γδ T cell compartments have undergone species-specific expansion and diversification, potentially driven by long-term adaptation to pathogen-rich environments and mucosal immune demands (33). In this context, the present study extends these findings by providing a direct comparative analysis of TRD repertoires between two closely related γδ T cell–high species. These findings provide a foundation for future functional investigations into γδ T cell–mediated immunity in ruminants, particularly in the context of pathogen recognition, vaccine responsiveness, and species-specific immune adaptation.

## Data Availability

The datasets presented in this study can be found in online repositories. The names of the repository/repositories and accession number(s) can be found below: https://www.ncbi.nlm.nih.gov/, PRJNA1417106.
